# Risk Factors of Intractable Epilepsy in Children with Cerebral Palsy

**DOI:** 10.22037/ijcn.v15i3.31556

**Published:** 2021

**Authors:** Yehia Hamed ABDEL MAKSOUD, Hany Abdelaziz SULIMAN, Sameh ElSAYED ABDULSAMEA, Naglaa MOHAMED KAMAL, Ashraf Hamed AL-SHOKRAY, Asmaa Obada IBRAHIM, Hatem Hamed ELSHORBAGY, Mohamed Gamal El Din FATHALLAH, Ahmed Mahmoud ATTIA, Akram Elshafey ELSADEK

**Affiliations:** 1 Pediatric department, Benha University, Benha, Egypt.; 2 Pediatric department, Faculty of medicine, Cairo University, Cairo, Egypt.; 3 Pediatric department, Ain shams University, Cairo, Egypt.; 4 Pediatric Department, Faculty of Medicine, Menoufia University, Shebeen Elkom, Egypt.; 5 Pediatric department, Qualiob General Hospital, Qaliob, Egypt.

**Keywords:** Risk factors, Intractable epilepsy, Cerebral palsy, Children

## Abstract

**Objectives:**

We aimed to investigate the risk factors predicting the development of intractable epilepsy in children with cerebral palsy (CP), with an emphasis on perinatal characteristics, seizure semiology, imaging, and EEG findings.

**Materials & Methods:**

Following a descriptive, retrospective, case-control design, 106 children with CP and epilepsy from 2015 to 2020 were studied (46 children with CP and intractable epilepsy and 60 with CP and controlled epilepsy). Data were retrieved from medical records of participants (i.e., demographics, clinical characteristics, perinatal history, etiology of seizure and CP, seizure semiology, intellectual functions, therapeutic options, brain imaging, and EEG findings).

**Results:**

We established a model of the most important risk factors that can predict intractable epilepsy in children with CP. The model included the additive effect of a poor Apgar score at 5 minutes, the presence of neonatal seizures, focal epilepsy, and focal slowing on the EEG background (Area under the receiver operating characteristic of 0.810).

**conclusion:**

The findings can be used to identify intractable epilepsy in children who suffer from CP with further support by offering early therapeutic interventions intended to reduce the burden of refractory seizures.

## Introduction

Cerebral palsy (CP) is a group of permanent, non-progressive disorders that affect muscle tone as well as movement or posture. It is caused by damages to the developing brain either before or during birth (1). Motor disorders of CP are often accompanied by epilepsy, disturbances of sensation, perception, communication, cognition, and behavior, as well as secondary musculoskeletal problems (2). 

It is well proved that epilepsy had a higher association with cerebral palsy; 15–60% of children with CP have been reported to be epileptics. The type of epilepsy among children with CP varies according to the type of CP (3). Previous studies not only had demonstrated the clinical characteristics and epidemiology of epilepsy in children with different types of CP (4, 5, 6), but also described its features, course, and outcomes, as well as consequences of refractory seizures (6). In some instances, refractory seizures are more problematic than the musculoskeletal complications of CP. 

The risk factors for the development of epilepsy later in life were commonly related to earlier onset of seizure and history of neonatal seizures (7). However, data related to birth conditions contribute to a lesser extent to the development of epilepsy in these patients (8). We aimed to explore the predictive factors that increase the risk of development of drug-resistant epilepsy in children with CP

## Materials & Methods

This descriptive, retrospective, case-control study was conducted on 106 children with CP and epilepsy (58 boys and 48 girls). The youngest and oldest participants were 3 months and 14 years old, respectively. Participants were selected among those referred to the pediatric neurology outpatient clinic of Al Hada, Taif military hospitals, Saudi Arabia, and Benha university hospital, Benha, Egypt, from 2015 to 2020. The study is approved by the institutional review board of the faculty of medicine of Taif University.

The exclusion criteria were:

(1) Children with CP who did not develop seizures;

 (2) Having neurodegenerative brain disease;

(3) Inborn errors of metabolism;

(4) Children with CP who had a history of isolated febrile seizures;

(5) Children with CP who had a history of isolated neonatal seizures with no seizures beyond the neonatal period.

(5) History of acute symptomatic seizures, defined as seizures at the time or proximity to a systemic or neurologic insult.

CP was defined as non-progressive encephalopathy that manifests as motor and postural disabilities. Epilepsy was defined as at least two unprovoked seizures with more than 24hr apart in the absence of acute insult.

Following the International Classification of Epilepsies and Seizure disorders, epilepsies were classified for all participants (9). A pediatric neurologist examined all children in order to approve their EEG. Brain imaging examinations were evaluated by a pediatric neurologist and neuroradiologist. Data on age, gender, age of onset of seizures, prenatal and perinatal history including gestational age, weight at birth, head circumference, Apgar scores at 1, 5, and 10 minutes, need for active resuscitation at the time of birth, and the history suggestive of hypoxic-ischemic encephalopathy, presence of neonatal seizures, history of epileptics, which was defined as a continuous seizure activity more than 30 minutes or recurrent attacks of seizures without regaining the conscious level in between (10), family history of epilepsy, history of delayed developmental milestones, EEG and brain imaging (brain CT/MRI), type, dose, and duration of antiepileptic drugs (AEDs), the response of seizure, refractory seizure, and any alternative management of seizures including ketogenic diet; vagal nerve stimulation; and epilepsy surgery, were retrieved from medical records of participants. Controlled epilepsy was defined as experiencing no seizure for more than 12 months, while drug-resistant epilepsy was defined as persistent seizures despite the proper use of at least two AEDs (proper selection- -optimum serum drug level, and good compliance)(11). Also, data related to CP included the type of CP, underlying cause, and growth motor function classification system (GMFCS) score.

Assessment of intelligent quotient (IQ) was done using neuropsychological tests. Mental retardation was defined as IQ < 70. Children with IQ < 35 were defined as severe mental retardation, 49–35 was considered as moderate mental retardation, and 69–50 was defined as mild mental retardation (12).

## Results

A total of 250 children with CP were recruited in the present study, of whom 130 were also suffering from epilepsy. Fourteen children were excluded from our study (2 had a neurodegenerative brain disease, one was suffering from an inborn error of metabolism, 3 had acute symptomatic seizures, 4 had isolated febrile seizures, and 4 had isolated neonatal seizures. As a result, data of 106 children with CP and epilepsy were analyzed.

Prematurity was found in 23% of participants. The most common type of CP was spastic quadriplegia (31.1%), followed by spastic hemiplegia (28.3%) and spastic diplegia (16.9%). However, spastic triplegia and extrapyramidal were reported in 11.3% and 7.5% of participants, respectively. Only 4.7% showed ataxic CP.

The most common cause of CP was prematurity (23%), followed by intracranial hemorrhage (20.7%) and cerebral infarction (15%). However, hypoxic-ischemic encephalopathy and central nervous system (CNS) infections were found in 13.3% and 11.3% of participants, respectively. About 10% of patients showed cerebral malformations, while 7.5% showed trauma as underlying etiology (Table 1). Generalized seizures, focal seizure, and focal onset seizure with secondary generalization were found in 41 (38.6.3%), 29 (27.3%), and 36 (33.9%) of participants, respectively. The age of onset of seizure was 6-40 months with a median of 19 months. The majority of cases were reported in the 1st year of life; (56.6% or n=60) cases. However, 2.8% (n=3) cases were diagnosed after the age of 10 years. Ninety-four cases (88.6 %) showed abnormalities in their EEG records. We found EEG abnormalities in the form of generalized (43 cases (40.5%)), focal (34 cases (32%)), and multifocal (12 cases (11.3%)) epileptogenic activities.

A specific EEG pattern was found in 4.7% (n=5) cases in the form of Hypsarrhythmia. However, abnormalities in EEG background included generalized slowing and focal slow rhythms in 37.7% (n=40) and 19.8% (n=21) participants, respectively. In addition, in the present study, intractable epilepsy was found in 43.3% (n=46) cases. The most common etiological factor responsible for focal-onset intractable epilepsy was intracranial Hemorrhage (22%), followed by cerebral infarction (19.5%) and CNS infection (13%). Nevertheless, brain malformation and trauma accounted for 8.7% and 4.3% of cases, respectively. The causes of CP leading to focal slowing on the EEG background in children with CP and intractable epilepsy were more or less similar, which included cerebral infarction (11%), intracranial bleeding (9%), CNS infection (4%), and, to a lesser extent, brain malformation (3%), and brain trauma (3%). Furthermore, we evaluated other variables that are reported as a probable consequence rather than a cause of intractable epilepsy. The following factors presented a significant association with intractable epilepsy: intellectual impairment (P =0.04), need for special education (P =0.02), a larger number of failed therapeutic trials (P =0.02), need for a ketogenic diet (P=0.001), history of undergone vagal nerve stimulation (P=0.03), and referral to epilepsy surgery (P=0.04). However, there was no significant association between the number of current AEDs and intractable epilepsy (P=0.43) (Table 1).

**Comparison of intractable epilepsy and controlled epilepsy in terms of association with independent risk factors**

Intractable epilepsy in children with CP was significantly associated with a low Apgar score (0-4) at 1 (P=0.03) and at 5 (P<0.0009) minutes as well as the need for active resuscitation at birth (P=0.02), Also, significant risk factors included younger age at the onset of the seizure (P=0.02), history of neonatal seizure (P=.003), history of status epilepticus as the initial presentation (P=0.04), focal-onset epilepsy (P < .001), and focal slowing on EEG record (Table 1). However, according to the findings, gender, prematurity, gestational age, body weight at birth, HIE, type CP, GMFCS score, etiology of CP, presence of a gross brain malformation, periventricular leukomalacia, IVH, microcephaly, type of epileptiform discharge, and positive family history of epilepsy did not show any significant changes between intractable epilepsy and controlled epilepsy (Table 1).

**Multivariate analysis of Predictors of intractable epilepsy:**

A low Apgar score (1-4) at 5 minutes was associated with increased risk of intractable epilepsy (by 3.17 times) (95% CI 1.41 -8.34, P = 0.03), while neonatal seizure was associated with 3.36 times increase in the risk of developing the condition (95% CI 1.36 -10.26, P =0 .02). However, for the focal onset Epilepsy and focal slowing in EEG background, the increase was equivalent to 3.43 (95% CI 1.81 -7.37, P =0 .008) and 4.35 folds (95% CI 1.48-16.8, P =0 .004) (Table 2), respectively.

We estimated the area under the receiver operating characteristic curve (AUC) for each variable, with further analysis of different combinations of the most significant variables (Table 3). The additions of all variables resulted in an AUC of 0.785. However, the addition of the 4 variables (i.e. a poor Apgar score of 1-4 at 5 minutes, development of neonatal seizures, focal epilepsy, and focal slowing on EEG background) resulted in the best model for the prediction of intractable epilepsy in children with CP, by yielding an AUC of 0.810 (Table 3). The additive effect of the independent variables that were more common in the intractable epilepsy group was also evaluated (Table 4).

**Table (1):** **Risk Factors for** **Intractable** **Epilepsy in Children with** **CP**

**Risk factor**	** Epilepsy**		**Total**	**P-value**
	**Intractable** (n =46)	**Controlled** (n =60)		
**Sex ** Male Female	28 (61) 18 (39)	33(55) 27(45)	61 (57.5) 45(42.5)	0.65
**Perinatal history** Gestational age, wk. <30 weeks 30-37 weeks > 37 **Birth weight, g(mean ± SD)** 500-1000 g 1000-1500 g 1500 -2000 g >2000 g **Apgar scores at minute 1** 0-4 5-7 8-10 **Apgar scores at minute 5** 0-4 5-7 8-10	32.6 ± 4.9 13(28) 12(26) 21 (45.6) 2346 ± 950 12(26) 7(15) 5(11) 22(48) 20(43.5) 12(26) 14(30.5) 18(39) 8(18) 20(43)	33.2 ± 6.8 22(36.6) 16(26.6) 22(36.6) 1938 ± 1075 7(11.6) 11(18.4) 12(20) 30(50) 10(16.7) 18(30) 32(53.3) 7(11.6) 10(16.7) 43(72)	32.9 ± 5.4 35(33) 28(26.5) 43(40.5) 2250 ± 1395 19(17.9) 18(16.9) 17 (16) 52(49) 30(28.3) 30(28.3) 46(43.4) 25(23.5) 18(16.9) 63(59.4)	0.42 0.37 0.41 0.36 0.28 0.64 0.54 0.27 0.45 0.03 0.18 0.34 0.0009 0.12 0;03
Active resuscitation at birth HIE Interventricular haemorrhage Periventricular leukomalacia Microcephaly	22(48) 11(24) 14(30) 9(20) 12(26)	11(18.4) 8(13.4) 19(31.6) 12(20) 10(16.6)	33(31.1) 19(17.9) 33(31.1) 21(19.8) 22(20.7)	0.02 0.14 0.73 0.28 0.57
**Cerebral palsy ** Type Spastic quadriplegia Spastic hemiplegia Spastic diplegia Spastic triplegia Extrapyramidal CP Ataxic CP	18(39.1) 13(28.2) 6(13.1) 4(8.7) 3(6.6) 2(4.3)	15(25) 17(28) 12(20) 8(13) 5(8) 3(5)	33(31.1) 30(28.3) 18(16.9) 12(11.3) 8(7.5) 5(4.7)	0.09
**GMFCS** 1 2 3 4 5 **Cause** Prematurity Intracranial haemorrhage(ICH) Cerebral infarction HIE CNS infection Brain anomalies Traumatic brain injury	10(22) 8(17) 6(13) 10(22) 12(26) 7(15.2) 10(22) 9(19.5) 8(17.3) 6(13) 4(8.7) 2(4.3)	13(22) 15(25) 10(17) 11(18) 11(18) 17(28) 12(20) 7(12) 6(10) 6(10) 6(10) 6(10)	23(21.6) 23(21.6) 16(15) 13(19.8) 23(21.6) 24(23) 22(20.7) 16(15) 14(13.3) 12(11.3) 10(9.4) 8(7.5)	0.36 0.19
**Epilepsy** Age at the onset of seizure; months (median (IQR)) History of neonatal seizures Positive family history of epilepsy Status epilepticus as initial presentation Type of seizure -Focal -Generalized -Focal with secondary generalization	14(2-42.5) 18(39.1) 4(8.7) 11(24) 22(48) 14(30) 10(22)	27(8-51.5) 7(12) 5(8) 7(11.6) 7(12) 27(45) 26(43)	19(6-40) 25(23.5) 9(8.4) 18(17) 29(27.3) 41(38.6) 36 (33.9)	0.02 003 0.15 0.04 0.0005
**Initial EEG record** **EEG background** ** activity** Focal slowing Generalized slowing **EEG activity and pattern** Generalized epileptogenic activity Focal epileptogenic activity Multifocal epileptogenic activity Hypsarrhythmia	14(30) 18(39.1) 11(24) 23(50) 10(22) 3(6.6)	7(12) 22(36.6) 32(53.3) 11(18) 2(3.3) 2(3.3)	21(19.8) 40 (37.7) 43 (40.5) 34 (32) 12(11.3) 5 (4.7)	0.02 0.04
Intellectual impairment Special education needs	36(78.2) 39(84.7)	26(43.3) 30(50)		0.04 0.02
Number of current medications (mean ± SD) Number of failed therapeutic trials (mean ± SD) Ketogenic diet Vagus nerve stimulation Referral for epilepsy surgery	3.2 ± 0.83 2.82±1.5 8(18) 3(6.6) 2(4.3)	1.6 ± 0.78 0.15 ± 0.43 0(0) 0(0) 0(0)	2.8±0.78 1.86±0.92 8(7.5) 3(2.8) 2(1.8)	0.43 0.02 0.001 0.03 0.04

**Table (2):** Multivariate Analysis of Risk Factors Predicting Intractable Epilepsy in Children with CP

**Clinical data**	**OR (95% CI)**	**P- value**
**Poor Apgar score at 5 minutes (1-4)**	3.17**(**1.41 -8.34)	0.03
**History of neonatal seizures**	3.36**(**1.36 -10.26**)**	0 .02
**Focal epilepsy**	3.43**(**1.81 -7.37**)**	0 .008
**Focal slowing on EEG** ** background**	4.35**(**1.48-16.8**)**	0 .004)

**Table (3):** Area under the Receiver Operating Characteristic Curve (AUC) for different variables predicting intractable Epilepsy

**Risk model**	**AUC – 1**
**Gestational age**	0.515
**Birth weight **	0.532
** Poor Apgar score (1-4) at 1 min **	0.785
**Poor Apgar score (1-4) at 5 min** *	0.625
**Need of active resuscitation at birth**	0.537
**HIE**	0.546
**Microcephaly**	0.573
**Brain** ** anomalies**	0.572
Younger age of seizure onset*	0.628
**Development of neonatal seizures**	0.563
**Status epilepticus as initial presentation**	0.545
**Focal epilepsy** *	0.627
Focal slowing of EEG background*	0.644
**Combination of all variables**	**0.785**
Combination of all the variables*	**0.810**

**Table (4):** **Probabilities of Epilepsy Being** **intractable** **in Children** **with CP with Different Combinations of** **Predictive Variables**

**Poor Apgar** **score at** **5 min**	**Neonatal** **seizures**	**Focal** **epilepsy**	**Focal** **slowing** **of EEG background**	**Number of** **risk factors** **present**	**Probability**
**Yes**	**Yes**	**Yes**	**Yes**	**4**	**0.87**
**No**	**No**	**No**	**No**	**0**	**0.04**
**Yes**	**No**	**No**	**No**	**1**	**0.13**
**No**	**Yes**	**No**	**No**	**1**	**0.16**
**No**	**No**	**Yes**	**No**	**1**	**0.17**
**No**	**No**	**No**	**Yes**	**1**	**0.18**
**Yes**	**Yes**	**No**	**No**	**2**	**0.28**
**No**	**No**	**Yes**	**Yes**	**2**	**0.32**
**Yes**	**No**	**Yes**	**No**	**2**	**0.36**
**Yes**	**No**	**No**	**Yes**	**2**	**0.40**
**No**	**Yes**	**No**	**Yes**	**2**	**0.43**
**No**	**Yes**	**Yes**	**No**	**2**	**0.47**
**Yes**	**Yes**	**Yes**	**No**	**3**	**0.58**
**Yes**	**Yes**	**No**	**Yes**	**3**	**0.64**
**Yes**	**No**	**Yes**	**Yes**	**3**	**0.66**
**No**	**Yes**	**Yes**	**Yes**	**3**	**0.74**

## Discussion

In this retrospective study, we studied epileptic children with CP according to demographic data, clinical characteristics, perinatal history, neuroimaging features, and etiological factors to investigate the risk factors that can predict the development of intractable epilepsy.

Refractory seizure not only causes significant morbidity and mortality but also has a high psychosocial burden with many implications in children with CP (13). The present study aimed to investigate factors that can predict intractable epilepsy in children with CP. According to the findings, poor Apgar score at 5 minutes, neonatal seizures, focal epilepsy, and focal slowing of EEG background were more significant than other factors. Based on the effect of these independent factors, we established a prediction model. The accuracy of the model was validated by AUC, which expressed good discriminatory power. Further analysis showed that the probability of intractable epilepsy is high in cases that have all these variables together. However, the combinations of some of these variables did not necessarily yield a high probability of intractable epilepsy. In our study, the incidence of epilepsy and intractable seizures in children with CP were comparable to those reported by other authors (14, 15, 16, 17, 18). In the present study, we defined intractable epilepsy according to the ILAE commission consensus proposal: “failure of a therapeutic trial of two maximally tolerated, appropriately chosen and properly used AEDs”, contrary to other studies that used a time frame of seizure-free period or did not consider a time reference at all (11). A unique finding of the present study is the positive correlation between a poor Apgar score and intractable epilepsy in children with CP. It is known that prenatal and perinatal problems are the major causes of CP in children. However, sufficient evidence is not available about the effect of these factors on the development of intractable epilepsy. Hence, in the present study, we determined the detailed data of prenatal and postnatal problems. Few studies mentioned a positive association between birth conditions and prognosis of epilepsy in children with CP (19). However, a poor Apgar score is associated with an increased risk of developing epilepsy in the general population and for those who suffer from CP in particular (20, 21, 22). Previous studies mentioned the relation between gestational age or birth weight and the development of epilepsy in children with CP. However, the results are conflicting. The association between gestational age and birth weight with intractable epilepsy was lacking in our study; however, there are studies that support our findings (18, 19). According to the findings, prematurity and low birth weight were not related to the risk of developing epilepsy (18). Kulak et al. found an increased risk of epilepsy in patients with low birth weight, but they did not reveal any association between gestational age and the risk of epilepsy development (23). Zelnik et al. reported that epilepsy was more prevalent in full-term infants than in preterm; meanwhile, they found no association between birth weight and increased risk of epilepsy development (6). In 17 European registers, some authors reported an association between epilepsy development and term and having a weight of ≥ 2500 g (15). Also, it has been reported that term delivery had a significant association with epilepsy development (24). These findings can be attributed to the fact that epilepsy usually stems from gray matter lesions, which are more prevalent in full-term babies, while white matter lesions are more common in premature infants (25, 26). The increased risk of epilepsy in children with CP has been attributed to genetic and perinatal factors (4). Among the perinatal factors, brain anomalies, chromosomal defects, intrauterine infections, and neonatal HIE are the more obvious causes that may result in seizures. Brain imaging may provide a diagnostic clue to the timing and nature of the brain insult in these children (5). In our study, the type of CP or its etiology or the GMFCS score did not show a significant association with intractable epilepsy. Regarding the type of CP, some authors reported similar findings, while others found inconsistent results. The difference can be attributed to the applied methodologies (18, 19). The frequency of epilepsy varies according to the CP subtype. According to the literature, epilepsy is particularly more common in patients with quadriplegic CP, while it is less common in children with dyskinetic or ataxic CP (4, 6, 24). The etiology of CP was classified according to the findings of brain imaging. It was found that children with evidence of CNS infection or brain anomalies in their brain imaging had a poor prognosis regarding seizure control compared to those with white matter injury or idiopathic causes (27). Despite the positive association between epilepsy in general and having a higher GMFCS score, our results showed an insignificant association between GMFCS score and intractable epilepsy. Contrary to our findings, Mert et al. reported an insignificant impact of GMFCS score on epilepsy prognosis (18, 19, 28). This can be attributed to the fact that GMFCS is a score that reflects motor system affection caused by white matter injury. Previously, it was reported that a history of neonatal seizures in children with CP is a risk factor for later epilepsy development (15, 18, 23, 24). Going with the current evidence of epilepsy in children with CP, we found that younger age at onset of the seizure, especially neonatal-onset seizure, was associated with intractable epilepsy (29,30). Therefore, we concluded that neonatal seizure, especially during the first 72 h, was a major risk factor for the prediction of later development of intractable epilepsy. The Collaborative Perinatal Project (NCPP) of the NIH reported a strong association between neonatal seizures and epilepsy (31). Some studies provided evidence that neonatal seizures can predict the future development of epilepsy in children with CP (32, 33). The history of neonatal seizures in children with CP is a risk factor for epilepsy development. Subsequently, the outcome for seizure control was negatively affected by this history, and patients with a history of neonatal seizures are at increased risk of intractable epilepsy prognosis compared to those without such a history (18). In our study, most children with CP had generalized seizures, as reported in previous studies (17, 18, 23, 24, 34). This can be attributed to the high number of children who suffered from spastic quadriplegic CP with diffuse brain injury in our sample of patients. There are many controversies regarding our finding that focal seizures were associated with intractable epilepsy. While some studies report similar findings (35, 36), others found contradictory results(18). For instance, it has been reported that focal seizures are one of the most important predictive factors of the worst outcomes of overall epilepsy in children (36). The strong association between intractable epilepsy and focal seizure can be attributed to its association with focal structural brain lesions (37, 38). This association was highlighted in our study, which showed all causes of CP that lead to focal epilepsy could be caused by focal brain pathology. Also, we observed a significant association between SE and intractable epilepsy, which is supported by other studies that reported a higher incidence of SE in children with CP and considered SE as an independent risk factor for intractable epilepsy (14, 30). This association can be explained by the fact that SE leads to brain damage (39). We reported changes in the EEG records in our cohort of patients in the form of generalized background slowing and focal activity. These EEG changes have been corroborated by several studies (4, 16, 23, 34, 35). In our study, EEG changes that are predictive of intractable epilepsy were focal, multifocal discharges, and focal background slowing. These EEG changes are an indication of intractable epilepsy in epileptic children (43, 44). Indeed, evidence regarding the predictive value of EEG on intractable epilepsy in children with CP are not sufficient (19). Mert et.al. reported that the presence of definite epileptogenic activity in EEG is significantly associated with more severe outcomes. However, their study suffers the limitation of describing either the type of epileptogenic activity or background rhythm abnormalities (18). Also, the pattern of hypsarrhythmia in EEG is a poor prognostic factor (23). The focal slowing in the EEG background may indicate a focal destructive lesion within the white matter of the brain of whatever the etiology. The etiological factors include trauma, brain anomalies, vascular lesions, and CNS infections that may lead to the generation of epileptic focus and later to cerebral dysfunction. According to the findings, these etiologies have been reported mainly in children with CP and epilepsy with a focal slowing in the EEG background. Our results are in line with studies that described focal EEG changes in children with hemiplegic CP (43, 44). There is a link between EEG abnormalities and behavioral and cognitive impairment in children with CP (4). That is why we recommend EEG recording on a routine basis for follow-up of children with CP. Children with spastic quadriplegia and hemiplegia experience developmental delay and intellectual disability, which matches brain damage commonly found in these forms of CP. Furthermore, it was significantly associated with intractable epilepsy. Indeed, the association between CP, epilepsy, and cognitive impairment, has been declared to be significant (4, 15). Mert et al. mentioned mental retardation as a risk factor for epilepsy in children with CP (16,18). Other authors found no significant difference between CP and epilepsy in terms of the social quotient, despite the fact that seizures are more frequent and intractable in patients with mental retardation. It is difficult to determine that cognitive impairment is either a cause or a resultant effect of intractable epilepsy. Therefore, we did not include cognitive impairment as a risk factor in the prediction model of intractable epilepsy (45, 46).

## Limitations

It is necessary to mention some limitations and biases of our study, including following a retrospective design, the heterogeneity of the etiological factors of CP, and epilepsy. These limitations probably have declined the accuracy of conclusions regarding the characteristics of intractable epilepsy. Further prospective studies on homogeneous groups, classified according to etiology and imaging findings, are recommended.

## In Conclusion

According to the findings, we can conclude that the most important risk factors that can predict intractable epilepsy in children with CP include poor Apgar score at 5 minutes, presence of neonatal seizures, focal epilepsy, and focal slowing on the EEG background. Intractable epilepsy may cause major adverse medical and psychosocial consequences. Our results can help clinicians and neurologists for the identification of children with CP and those at high risk of epilepsy, with further support to offer early therapeutic interventions intended to reduce the burden of refractory seizures.
